# An Effective Support System of Emergency Medical Services With Tablet Computers

**DOI:** 10.2196/mhealth.3293

**Published:** 2015-02-27

**Authors:** Kosuke C Yamada, Satoshi Inoue, Yuichiro Sakamoto

**Affiliations:** ^1^Division of Trauma Surgery and Surgical Critical CareFaculty of MedicineSaga UniversitySagaJapan; ^2^Department of Emergency MedicineFaculty of MedicineSaga UniversitySagaJapan

**Keywords:** emergency medical services, EMS, EMS communication systems, prehospital, ambulance, tablet computers, cloud computing

## Abstract

**Background:**

There were over 5,000,000 ambulance dispatches during 2010 in Japan, and the time for transportation has been increasing, it took over 37 minutes from dispatch to the hospitals. A way to reduce transportation time by ambulance is to shorten the time of searching for an appropriate facility/hospital during the prehospital phase. Although the information system of medical institutions and emergency medical service (EMS) was established in 2003 in Saga Prefecture, Japan, it has not been utilized efficiently. The Saga Prefectural Government renewed the previous system in an effort to make it the real-time support system that can efficiently manage emergency demand and acceptance for the first time in Japan in April 2011.

**Objective:**

The objective of this study was to evaluate if the new system promotes efficient emergency transportation for critically ill patients and provides valuable epidemiological data.

**Methods:**

The new system has provided both emergency personnel in the ambulance, or at the scene, and the medical staff in each hospital to be able to share up-to-date information about available hospitals by means of cloud computing. All 55 ambulances in Saga are equipped with tablet computers through third generation/long term evolution networks. When the emergency personnel arrive on the scene and discern the type of patient’s illness, they can search for an appropriate facility/hospital with their tablet computer based on the patient’s symptoms and available medical specialists. Data were collected prospectively over a three-year period from April 1, 2011 to March 31, 2013.

**Results:**

The transportation time by ambulance in Saga was shortened for the first time since the statistics were first kept in 1999; the mean time was 34.3 minutes in 2010 (based on administrative statistics) and 33.9 minutes (95% CI 33.6-34.1) in 2011. The ratio of transportation to the tertiary care facilities in Saga has decreased by 3.12% from the year before, 32.7% in 2010 (regional average) and 29.58% (9085/30,709) in 2011. The system entry completion rate by the emergency personnel was 100.00% (93,110/93,110) and by the medical staff was 46.11% (14,159/30,709) to 47.57% (14,639/30,772) over a three-year period. Finally, the new system reduced the operational costs by 40,000,000 yen (about $400,000 US dollars) a year.

**Conclusions:**

The transportation time by ambulance was shorter following the implementation of the tablet computer in the current support system of EMS in Saga Prefecture, Japan. The cloud computing reduced the cost of the EMS system.

##  Introduction

### The Ageing Society of Japan

Japan is a world’s fastest aging society [1]. According to the Cabinet Office, Government of Japan, people age 65 and over are estimated to reach 3,657,000 (the percentage of the population age 65 and over, 30.3%) in 2025 [2]. Changing demographics caused by the aging population have been increasing concern about longevity crisis, especially the national burden of long-term care insurance costs in Japan. All regions in Japan have been urged to deal effectively with the aging of the population. Now the efforts of Japan are focus of other societies.

In the Japanese emergency medical services (EMS), like the aging society, the increase in ambulance dispatches and the transportation time by ambulance has become a significant social problem. There were over 5.4 million ambulance dispatches during 2010 in Japan, and the time for transportation is increasing with a national average of 37.4 minutes [3]. As with all regions in Japan, Saga Prefecture, the northwest part of the island of Kyushu (population 850,000), has the problems of increased time of transportation, an increased number of emergency transports, and a decreased number of emergency facilities. In Saga, the number of emergency transports has increased from about 22,000 cases in 2000 to about 30,000 cases in 2010 and the transportation time has also gradually increased with an average of 35.5 minutes [3].

In Japan, emergency transport service is controlled and managed by each local government, and Japanese emergency personnel; for example, paramedics belong to the fire department as public civil servants [4]. Although Japanese emergency personnel are nationally licensed, the law restricted their medical practices. Their main role at the scene is to make triage and destination decisions. When the emergency personnel select a destination to transport the patients from the scene, their experience and knowledge in the past and information of each local fire department are matched to an appropriate hospital in their community. Unfortunately, it frequently occurs that it takes time to select a destination to transport due to reasons such as all beds occupied and doctors out of their specialty.

### Levels of Emergency Medical Services

There are three levels of EMS classified by function and scale in Japan [4,5]. Primary care facilities represent small outpatient clinics without beds that deal with patients with relatively mild symptoms and do not necessarily require emergency medical treatment or hospitalization. Secondary care facilities are capable of providing more advanced patient care, including operations and other interventions requiring hospitalization for patients with serious illnesses. Tertiary care facilities are medical emergency centers for critically ill patients who need intensive care or emergency surgery. In Saga, the number of tertiary care facilities has decreased from about 64 in 2000 to 48 in 2010, while the number of emergency cases is increasing [3,6]. Therefore, the required amount of transportation to tertiary care facilities has increased from 27.5% in 2007 to 32.7% in 2010 [3,6]. A solution to reduction of the transportation time by ambulance is to shorten the time of searching for an appropriate facility/hospital during prehospital phase.

### Telemedicine

Evidence of beneficial telemedicine was demonstrated in other medical fields [7-9], and studies have examined information sharing via the Internet or telecommunication devices throughout the world [10-14]. For instance, in the management of stroke cases, a system using a mobile device in which shared data and the diagnostic image of the patient has developed, and the system was shown to be efficient for early patient treatment [10]. Bouri and Ravi [11] reviewed previous researches regarding mobile health, and suggested the potential benefits and challenges of telecommunication with mobile devices in emergency response in the United States. Most of EMS systems in previous studies are to transmit electrocardiogram [14-19] and images [10,13,18-20] from the scene or an ambulance to the hospital. In the EMS, to send “the right thing in the right way to the right patient at the right time”[21] is the most crucial to critically ill patients. Although the telemedicine of transmitting electrocardiogram and images is useful, it is more significant for EMS to share information related to emergency medical demand and acceptance between the emergency medical personnel in the prehospital and the medical staff in the hospital.

To realize real-time information sharing during emergencies, we focused on cloud computing that has been receiving considerable attention by organizations. “Cloud computing is a model for enabling ubiquitous, convenient, on-demand network access to a shared pool of configurable computing resources (eg, networks, servers, storage, applications, and services) that can be rapidly provisioned and released with minimal management effort or service provider interaction” [22]. Cloud computing provides more flexible, cost-effective, and efficiency in information and communication technology (ICT) services to end users [23]. Previous studies expressed that cloud computing is a promising model with a big potential [24,25]. In recent years, researches showed many benefits of cloud computing for health care fields, especially the biomedical informatics community, which has already demonstrated good use of cloud computing [23,26-28]. As with these studies, several other researches suggested that they could improve emergency health care service [11-14,23]. Thus, it should serve the purpose to share information related to emergency medical demand and acceptance between prehospital and in-hospital.

The conventional hospital searching system for EMS in Saga Prefecture was seldom utilized, mainly because data was unavailable from the ambulance without Internet access. In the old system, some data could be shared only among the hospitals, but not from an ambulance, creating obstacles to obtain the latest information due to limited server capacity and slow Internet connection. In an attempt to improve the drawbacks of the previous system, we established a new system that is able to obtain the latest information regarding emergency demand and acceptance in one view using a tablet computer [18]. The focus of the new system was to share information related to emergency demand and acceptance.

The purpose of this study is to evaluate if the new system promotes efficient emergency transportation for critically ill patients. The following was examined to: compare the data for mean transportation times in Saga Prefecture before and after introducing a new system, compare them to nationwide trends observed over the same time period, examine the utilization of a new system by a user of emergency personnel and the medical staff, and, finally, investigate the cost effect of introduction of a new system.

## Methods

### System Function and Operation

**Figure 1:**
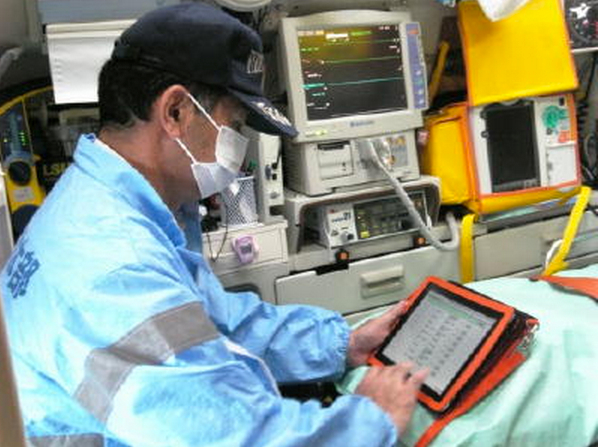
The emergency medical service (EMS) personnel handling with the tablet computer to check the status of patients’ transfer.

The new system to check emergency demand and acceptance adopted cloud computing so that both emergency personnel in the ambulance and the medical staff in each hospital are able to share up-to-date information about available hospitals. While the emergency personnel usually operate the application outside of the hospital, the medical staff mainly operates it with the desktop computer in the hospital. The Web application is developed and is compatible with both desktop/laptop computers and tablet devices. All of the operations on any device are run through a Web browser. All 55 ambulances in Saga Prefecture are equipped with tablet computers continuously connected with third generation (3G)/long term evolution (LTE) networks (Figure 1 shows this).

**Figure 2:**
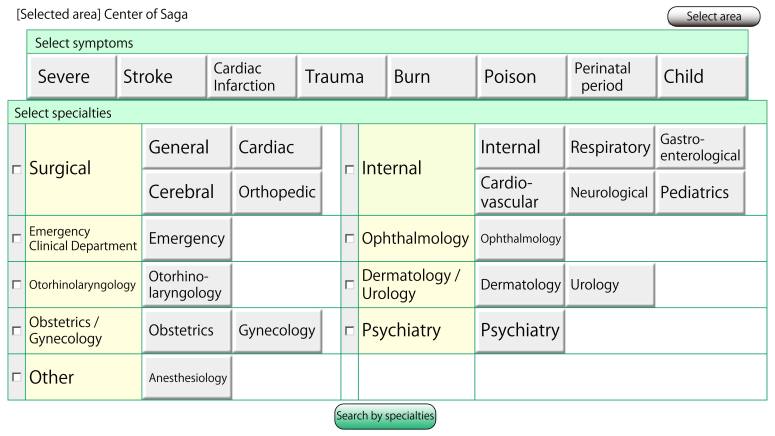
Search page to find available hospitals.

**Figure 3:**
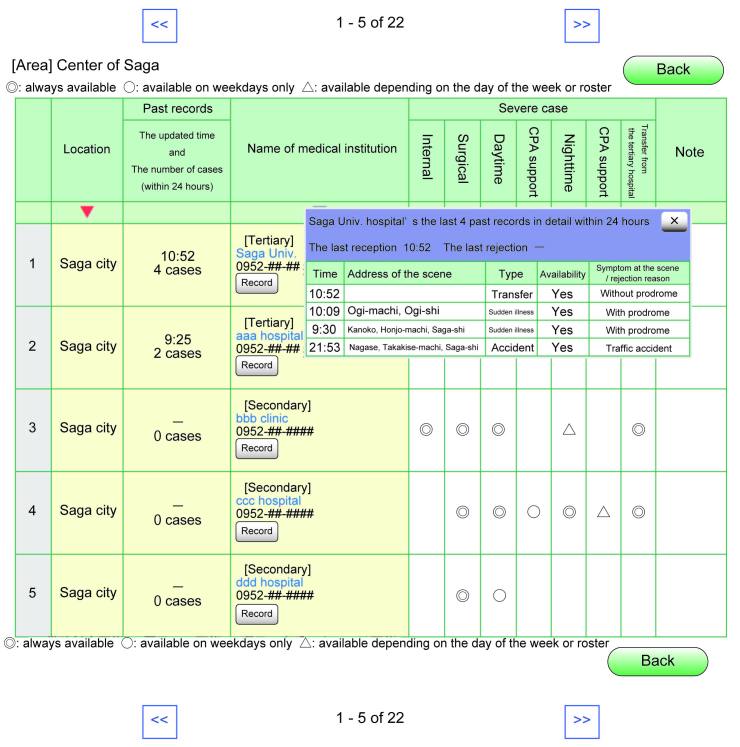
A search result page for severe cases.

When the emergency personnel arrive on the scene and discern the type of patient’s illness, he or she can open the application on their tablet computer to search for an appropriate facility/hospital based on specific conditions (eg, severity, trauma, burn) and specialties (Figure 2 shows this). The system then shows the list of available facilities (Figure 3 shows this). The search result includes the following information: the updated time and date, the number of patients each hospital accepted over the last 24 hours and the last time of acceptance, the telephone number of the facilities, the availability of each hospital with available specialties (subdivided into always available, available on weekdays only, available depending on the day of the week or roster), and detailed information of the last five transportation records within 24 hours (time of day, the address of the scene, condition of the patient, accepted or denied by facilities, the mechanism or cause of the disease and injury at the scene). Facilities that do not update the information about their availability are moved to the bottom of the list.

**Figure 4:**
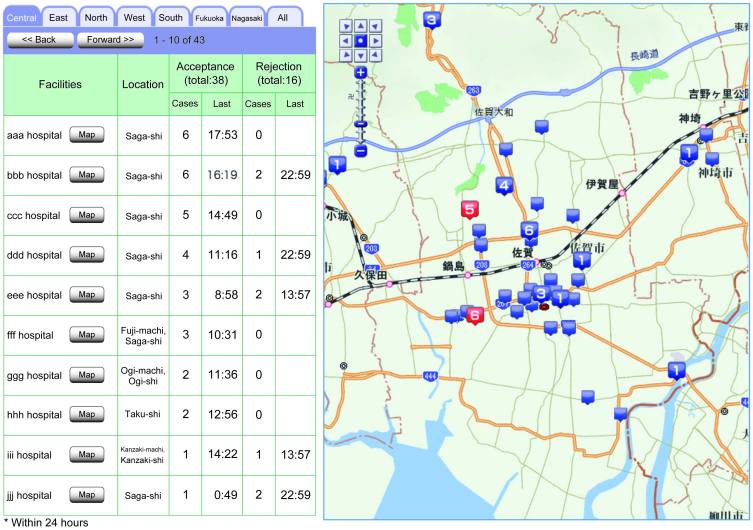
Real-time monitoring page with a map.

In addition, the new system visualizes the real-time monitoring of the number of acceptances in each hospital within 24 hours on a map so medical personnel can easily recognize the current status of each hospital located within and surrounding the region of Saga Prefecture (Figure 4 shows this).

The emergency personnel and medical staff input data for each case to the database in the new system through their tablet computer either in the ambulance on their way back to the fire department or in the hospital after transportation. On the other hand, the medical staff enters data about the availability of their hospital two or three times in the morning and evening. Since facilities that accepted emergency patients have to input data on the outcome of each patient, the new system includes information not only about the status of transportation by ambulance in each area, but about a final outcome including types of injury and disease.

### Measures

Data including the following four measurements were collected prospectively between April 1, 2011 and March 31, 2013.

#### Transportation Time in Minutes

Transportation time was defined as the time required for transporting from emergency “119” calls to arrival to the hospital. This measure is expressed in the mean, and commonly used as an indicator representing the conditions of present EMS in Japan [3].

#### Ratio of Transportation to the Tertiary Care Facilities as a Percentage

Ratio of transportation to the tertiary care facilities was calculated by dividing cases of transportation to the tertiary care facilities by all cases of transportation.

#### System Entry Completion Ratio as a Percentage

When the entering of all the data into the system is completed, the system automatically records flag information. For the emergency personnel, flag information is recorded when they input all the data such as date, types of the incident, gender and age group of patients, and time of day for receiving 119 calls or arriving at hospitals for each case to the database in the system. For the medical staff, flag information is recorded when they input data on the outcome of each patient. The system entry completion ratio was calculated by dividing flagged cases of transportation by all cases of transportation for both the emergency personnel and the medical staff.

#### Operational Costs

Although the Saga Prefectural Government traditionally provided the operation of the old system, the operation of the new system was entrusted to the private sector. The company entrusted with operation provided cloud computing services. All the Saga Prefectural Government has to do is to pay the rate for the service corresponding to the payment ratio. The operational costs in this article were a service charge to be paid by the Saga Prefectural Government to the entrusted private sector.

## Results

### Number of Transportations

Over a three-year period, the number of transportations by ambulance in Saga was 30,709, 30,772, and 31,629, in 2011, 2012, and 2013, respectively. All the data were cleaned and analyzed in this article.

### Transportation Time in Minutes

**Figure 5:**
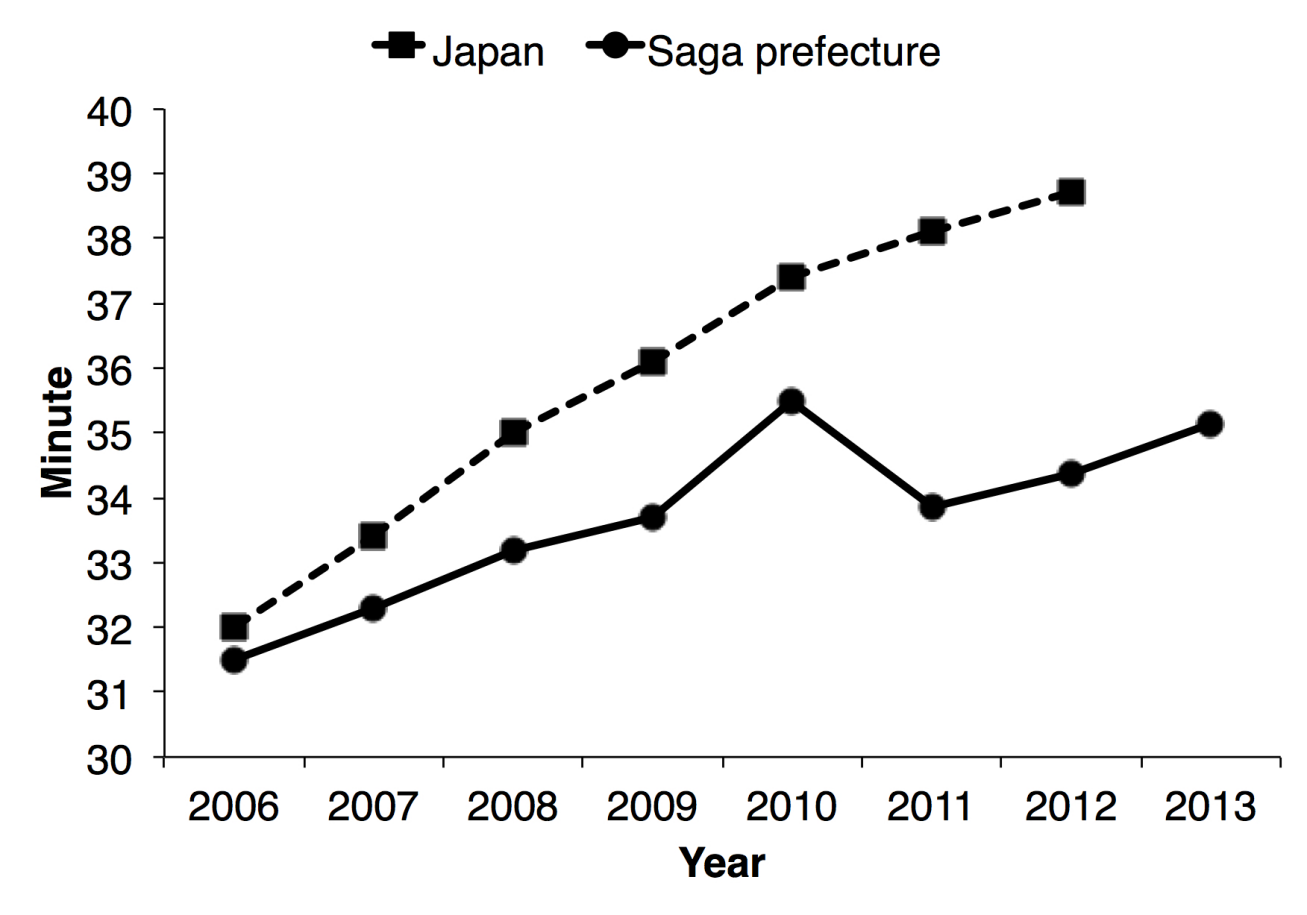
Mean transportation time in minutes in all of Japan and Saga Prefecture.

In Saga, the transportation time by ambulance was shortened for the first time since the statistics were first kept in 1999. The transportation time was 35.5 minutes (min) in 2010 (national average) and 33.9 min (95% CI 33.6-34.1 min) in 2011 (Figure 5 show this). Then it prolonged gradually 34.4 min in 2012 (95% CI 34.1-34.6 min) and 35.1 min in 2013 (95% CI 34.9-35.4 min). Although nationwide trends of the transportation time in Japan continued to expand, that in Saga was temporarily shortened in 2011, and was lower than that in Japan.

### Ratio of Transportation to the Tertiary Care Facilities in a Percentage

The ratio of transportation to the tertiary care facilities in Saga has decreased by 3.12% from the year before, 32.7% in 2010 (regional average) and 29.58% (9085/30,709) in 2011. Then that has slightly decreased to 29.53% (9088/30,772) in 2012 and 28.86% (9127/31,629) in 2013.

### System Entry Completion Rate in a Percentage

The system entry completion rate entered by the emergency personnel was 100.00% (93,110/93,110) over the observational time period from 2011 to 2013 (30,709/30,709 in 2011; 30,772/30,772 in 2012; 31,629/31,629 in 2013). The data on the transportation time by ambulance has been continuously stored in the system and data analysis is ongoing. The system entry completion rate entered by the medical staff was 46.11% (14,159/30,709), 47.57% (14,639/30,772) and 47.12% (14,905/31,629), in 2011, 2012, and 2013, respectively.

### Operational Costs

The new system reduced the operational costs by 40,000,000 yen (about $400,000 US dollars) in 2011. Although the previous system cost 67 million yen (about $670,000 US dollars) a year, the new system has kept a total annual cost for the cloud computing services less than 20 million yen (about $200,000 US dollars). There was seven million yen (about $70,000 US dollars) that was spent on the tablet computers to manage and operate them.

##  Discussion

### Benefits of the New System

Incorporation of this new system offered a number of positive effects on our emergency care system and reduced costs associated with the system. Particularly, the new system shortened transportation time by ambulance, which had been previously increasing every year. In addition, the ratio of transportation to the tertiary facilities was also reduced through the introduction of the new system. Finally, incorporation of the new system could get a serious cost reduction.

In the past, emergency personnel would have to make phone calls to nearby hospitals based on their experience and knowledge, or refer to the control center to find available hospitals for patients to be transferred. Therefore, neither the medical staff nor the emergency personnel could grasp the status as to which hospital is available to accept new patients and which one is accepting more or less emergency cases unless they check the information with each other. The introduction of this new system utilizes a mobile environment (3G/LTE networks) so that all medical personnel involved in prehospital patient care are able to check and share valuable information anytime and anywhere in real time. It is extremely useful that the emergency personnel can operate their tablets on the road or in/out of the hospital depending on the situation. The search result also shows the reasons that hospitals did not accept patients, such as fully occupied or an insufficient number of specialized doctors. It is imperative to apply suitable technology to the workflow of prehospital care right at the scene for efficient patient care and distribution to the appropriate hospitals. The introduction of this new system has enhanced information sharing among the emergency personnel and the medical staff in prehospital settings. This new system allowed the patients to be transferred to a suitable hospital for them and led to a decrease in the number of unnecessary transportations of mild cases to the tertiary facilities (level I trauma centers).

Very few EMS and medical staff utilized the old system. Even though some medical personnel input the information into the system, it was not certain whether the data was updated. Therefore, EMS was forced to contact a potential hospital by phone to obtain more reliable information. However, usage of the new system has significantly increased both in the hospitals (46 .11%, 14,159/30,709-47.57%, 14,639/30,772) and fire departments (100.00%, 30,709/30,709), compared with the previous system. Each member of the personnel can recognize updates on time and which hospital is neglecting to update in the new system. This motivates emergency personnel and medical staff to use the system and update the information more frequently because of a psychological effect provided by being evaluated and seen by others.

After the inception of the new system, it has successfully reduced the operational costs by 40 million yen ($400,000 US dollars) a year. The Saga Prefectural Government sought competing bids for running the new system. Total operational costs of the old system included the cost of power consumption of a server, the cost of the location of a server, the labor costs, and the cost for apparatus replacement. Cloud computing technology and services included above costs resulted in a significant cost reduction. Many studies also concluded that cloud computing is cost-effective [23-29]. The old system required many more expenses for purchasing and installation of the server and to maintain the operation and maintenance of the system. In contrast, cloud computing cut these costs, and its fees are on a pay-per-use basis. As a result, a 60% reduction in cost came from this new system [30]. The local government officers, to improve additional public services, could utilize the 40 million yen saved each year.

Unlike conventional emergency transfer support systems in Japan, the new system records not only the data of response rate to a demand, but also the clinical outcome after transportation. Therefore, the data of this new system enables us to review if an emergency patient is transferred to the appropriate hospital. Moreover, since each local government has their own regional arrangements and policies for EMS, they can amend the policies of their EMS using the regional data obtained from the new emergency support system.

### Limitations

This study has several limitations. While the new system provides the function of the real-time monitoring, data input timing differentiates information displayed on the real time monitoring page from the actual situation. For example, despite of the fact that a facility is available in search results and real-time monitoring, it actually is unavailable when the emergency personnel confirm the availability by call. It would be necessary to add and modify a function of displaying the course of transportation.

There are little scientific data to validate whether patients are transported to the appropriate hospitals. Although the ratio of transportation to the tertiary care facilities was decreased, it is obscure if the triage performed by the emergency personnel at the scene is suitable for selecting a destination to transport the patients. To improve quality of the medical care and public services, it would be essential information for the EMS system regarding the triage by the emergency personnel and diagnosis on arrival [31].

Last, data are insufficient to analyze evidence of the effects of transportation by ambulance on patient outcomes. In the new system, data of patient outcomes entered by the medical staff in hospitals are about the final result of patients at the hospital of a transport destination. If a patient is transferred to another hospital, the outcome of that patient is missing without an additional follow-up survey. It could be important for patient outcomes to create an integrated EMS system connected to electronic health records [32,33].

### Conclusions

This article reported that incorporation of ICT offered significant positive impact on the emergency medical setting. It is important for emergency personnel to share information about transportation records and available hospitals with the medical staff in real time for an effective EMS and appropriate patient distribution. Further studies are warranted to sufficiently identify the current state and issues of regional EMS to be improved by analyzing the recorded data in this new system.

## Abbreviations

3G: third generation
EMS: emergency medical services
ICT: information and communication technology
LTE: long term evolution
min: minutes
